# Effects of Growth Stages on Leaf Nutrients, Metabolites, and Polyphenols of Three Varieties of Haskap

**DOI:** 10.3390/foods13233762

**Published:** 2024-11-24

**Authors:** Chijioke Emenike, Shakirah Azeez, Marcia English, Chibuike Udenigwe, Lord Abbey

**Affiliations:** 1 Department of Plant, Food, and Environmental Sciences, Faculty of Agriculture, Dalhousie University, Truro, NS B2N5E3, Canada; labbey@dal.ca; 2 Faculty of Natural and Applied Sciences, Hezekiah University, Umudi, Nkwerre 471115, Nigeria; 3 Department of Food Science and Technology, Federal University of Technology, PMB 65, Minna 920101, Nigeria; 4 Department of Human Nutrition, Saint Francis Xavier University, Antigonish, NS B2G 2W5, Canada; menglish@stfx.ca; 5 School of Nutrition Sciences, University of Ottawa, Ottawa, ON K1N 6N5, Canada; cudenigw@uottawa.ca

**Keywords:** haskap leaves, berry nutrients, organic acids, amino acids, *Lonicera caerulea* L.

## Abstract

Haskap (*Lonicera caerulea* L.) has gained much research interest, given the diverse biologically active compounds found in different parts of the plant. It is, therefore, important to study the concentration of some of these biologically active compounds at different developmental stages. The present study investigated the effect of growth stages on nutrients, metabolites, and polyphenol concentrations in the leaves of three haskap varieties (Indigo Gem, Wojtek, and Jolanta). A targeted quantitative metabolomics approach was used to analyze the haskap leaves using a combination of direct injection mass spectrometry with a reverse LC-MS/MS custom assay. The results showed that the concentrations of soluble sugar, organic acids, polyphenols, and amino acids in the leaves of different varieties of haskap were decreased at the fruiting stage. The leafing stage may be the best period to harvest haskap leaves with the highest nutrients and polyphenol concentrations. Studies on polyphenols and nutrient characterization of haskap leaf at the leafing stage could be further investigated.

## 1. Introduction

Haskap (*Lonicera caerulea* L.) is a fruit-bearing shrub plant belonging to the Caprifoliaceae family. It is native to northern Japan, China, and southeastern Russia [1]. Haskap fruits have been used in folk medicine [2]. Over the past 10 years, *haskap* has received much research interest within Canada. Research studies have shown that haskap berries exhibit multiple health-promoting and therapeutic properties, including antioxidant, antimicrobial, anti-inflammatory, anti-diabetic, anti-obesity, and anticancer properties [3,4,5,6]. These have been attributed to the presence of phytochemicals such as anthocyanins, phenols, organic acids, and large amounts of vitamin C in the haskap berries [7,8,9].

Haskap is a perennial plant with a life span of up to 35 years that holds great promise as a source of additional raw materials [10]. It has been a relatively well-researched plant material in the last couple of years. However, most of the scientific research is focused on the berries, which are used in the food industry. Little to no particular attention is given to the rest of the plant. In a recent review by Cheng et al. [11], it was argued that not only does the berry fruit have nutritional values, but other parts of the plants, such as the flowers, leaves, and stems, also contain nutrients and biologically active compounds. Given the diverse biologically active compounds found in different parts of the plant, it is imperative to study the different untapped plant parts of haskap for the development of novel, natural-based bioproducts [12]. Furthermore, it is worth noting the growing trend of utilizing herbal infusions and the potential of haskap to complement pharmacotherapy in the management of diet-related ailments [13,14]. Therefore, it seems reasonable to explore the potential use of haskap berry leaves, as their collection does not impact the plant negatively [12]. Developing new applications for different parts of the plant could potentially create new raw materials for the food industry from what was previously considered waste.

During plant growth, several metabolic processes occur that might result in changes and/or redistribution of nutrient concentrations and biological profiles across the plant. This could be of importance to determine the best time of harvest for haskap leaves to maximize the total concentration of target compounds. This study aimed to investigate the effect of growth stages on the nutrient and polyphenol contents of three varieties of haskap leaf.

## 2. Materials and Methods

### 2.1. Location and Sample Collection

Leaves from three haskap varieties, namely Indigo Gem, Wojtek, and Jolanta, were collected at different growth stages (i.e., leafing, fruiting, and harvesting). Haskap leaves were collected from the North 45 Orchards Haskap Farm in Cloverdale, Stewiacke. Cloverdale is located at latitude 45.18° N, longitude −63.22° W. Leaf samples were picked from the 2nd leaf node from the tip of the stem; 30 leaves were sampled per plant from 5 plants per planting row in triplicate. The haskap leaves were wrapped in aluminum foil and placed in a Styrofoam pack containing liquid nitrogen before being transported to the plant physiology laboratory in the Department of Plant, Food, and Environmental Sciences, Dalhousie University. The leaves were ground in liquid nitrogen using a mortar and pestle and stored in 50 mL centrifuge tubes at −80 °C until further analysis.

### 2.2. Chemicals

Formic acid, methanol, acetonitrile, and water (LC/MS grade) were purchased from Optima™, and phenol, D-glucose, sulphuric acid, and ammonium acetate (LC/MS grade) were purchased from Fisher Scientific. HPLC grade pyridine, chloroform, methyl tert-butyl ether, hexane and ethanol, phenylisothiocyanate (PITC), 3-nitrophenylhydrazines (3-NPH), 1-ethyl-3-(3-dimethyl aminopropyl) carbodiimide (EDC), and butylated hydroxytoluene (BHT) were purchased from Sigma-Aldrich (St. Louis, MO, USA). All chemical standards and the corresponding isotope-labeled internal standards were purchased from C/D/N Isotopes Inc. (Pointe-Claire, CA, USA).

### 2.3. Soluble Sugar Determination

The soluble sugar of the different varieties of haskap leaf was analyzed at the leafing, fruiting, and harvesting growth stages, according to the method described by Wang et al. [15] with modification. One gram of haskap leaf powder was measured in a 15 mL Eppendorf tube, and 10 mL of 90% ethanol was added and vortexed for 30 secs. The tube was sealed and incubated in a water bath at 60 °C for 1 h. The solution was made up to a final volume of 25 mL by adding 90% ethanol, vortexed, and centrifuged at 4000× *g* for 3 min. One milliliter of aliquot was transferred into a thick-walled glass test tube, and 1 mL of 5% phenol was carefully added and mixed thoroughly. Five milliliters of concentrated sulphuric acid was added and mixed thoroughly. The mixture was then incubated in a dark space for 15 min. After that, it cooled to room temperature. The absorbance was measured at an absorbance of 490 nm using a UV–vis spectrophotometer (Jenway, Staffordshire, UK) against a blank containing deionized water, phenol, and sulphuric. The soluble sugar concentration was calculated using a standard calibration curve.

### 2.4. Metabolite Analysis

Samples of 25 mg were thawed on ice and were vortexed and centrifuged at 13,000× *g*. A quantity of 10 μL of each sample was loaded onto the center of the filter on the upper 96-well plate and dried in a stream of nitrogen. Subsequently, phenyl-isothiocyanate was added for derivatization [16]. After incubation, the filter spots were dried again using an evaporator. The metabolite extraction was then achieved by adding 300 μL of extraction solvent. The extracts were obtained by centrifugation into the lower 96-deep well plate, followed by a dilution step with MS running solvent. Data were collected using a TMIC PRIME Plant assay using DI/LC-MS/MS and a polyphenol assay using LC-MS/MS. A targeted quantitative metabolomics approach was used to analyze the samples using a combination of direct injection mass spectrometry with a reverse-phase LC-MS/MS custom assay with an ABSciex 4000 QTrap (Applied Biosystems/MDS Sciex, Foster City, CA, USA) mass spectrometer [16]. The method combines the derivatization and extraction of analytes and selective mass-spectrometric detection using multiple reaction monitoring (MRM) pairs. Isotope-labeled internal standards and other internal standards were used for metabolite quantification. Mass spectrometric analysis was performed on an ABSciex 4000 Qtrap^®^ tandem mass spectrometry instrument (Applied Biosystems/MDS Analytical Technologies, Foster City, CA, USA) equipped with an Agilent 1260 series UHPLC system (Agilent Technologies, Palo Alto, CA, USA). The samples were delivered to the mass spectrometer by an LC method followed by a direct injection (DI) method.

### 2.5. Polyphenol Analysis

Samples of 7.5 mg (dry weight) of leaves were weighed accurately into 1.5 mL microcentrifuge tubes, followed by the addition of 10 μL of a mixture of ten isotopic-labeled ISTDS and 250 μL of 80% methanol in water (*v*/*v*). Each tube was then vortexed for 30 min and spun down for 5 min at 14,000× *g*. After that, the supernatant from each tube was transferred and filtered through a 0.22 μm Acrodisc^®^ syringe filter (Pall Corporation, New York, NY, USA) with a GHP membrane for UHPLC-HRMS analyses. Solvent A: 0.1% (*v*/*v*) formic acid in water; Solvent B: 0.1% (*v*/*v*) formic acid in methanol. The chromatographic separation of polyphenols was achieved using an 8 min LC gradient method, and the LC column chamber was maintained at 30 °C. The flow rate was 500 μL/min, and the sample injection volume was 5 μL. Data acquisition was carried out using parallel reaction monitoring in negative ionization mode, and analyte peak identification followed by peak integration was performed using Trace Finder 4.1 software. The sample sequence included quality control samples at three different concentrations (low, mid, and high) and calibration solutions at eight different concentration levels. The ratios of the signal intensity of each analyte to its corresponding ISTD were plotted against the known concentrations of the standard mixtures to build the calibration curves [17]. A Thermo Scientific Vanquish UHPLC system (Thermo Scientific, Waltham, MA, USA) and an Agilent reversed-phase Zorbax Eclipse XDB C18 column (3.0 mm × 100 mm, 3.5 μm particle size, 80 Å pore size) with an ACQUITY UPLC CSH C18 VanGuard Pre-column (2.1 mm × 5 mm, 1.7 μm particle size, 130 Å pore size) were used for all online UHPLC-HRMS analyses with a Thermo Scientific Q-Exactive HF Hybrid Quadrupole-Orbitrap Mass Spectrometer (Waltham, MA, USA). The controlling software for the sample analysis was Thermo Xcalibur 4.3.

### 2.6. Organic Acid Analysis

A quantity of 150 μL of ice-cold methanol and 10 μL of an isotope-labeled internal standard mixture were added to 50 μL of each leaf sample extract for overnight protein precipitation. Then, it was centrifuged at 13,000× *g* for 20 min before 50 μL of the supernatant was loaded into the central wells of a 96-well deep well plate, followed by the addition of 3-nitrophenylhydrazine (NPH) reagent. After incubation for 2 h, BHT stabilizer and water were added before LC-MS injection [17].

### 2.7. Statistical Analysis

Data for polyphenols, amino acids, and organic acids were analyzed using Analyst 1.6.3 [17]. Data from soluble sugar were analyzed using analysis of variance. Means with significant differences at *p* < 0.05 were separated using Turkey’s LSD methods (Minitab, Inc., State College, PA, USA). Additionally, Pearson correlation matrix analysis, heatmap correlation analysis, partial least squares (PLS) regression analysis, and principal component analysis (PCA) were conducted using XLSTAT version 2022.3 (Addinsoft, New York, NY, USA). Graphs and pie charts were plotted using GraphPad Prism 9 Software (USA).

## 3. Results

### 3.1. Soluble Sugar (SS) in Haskap Leaves

**Figure 1:**
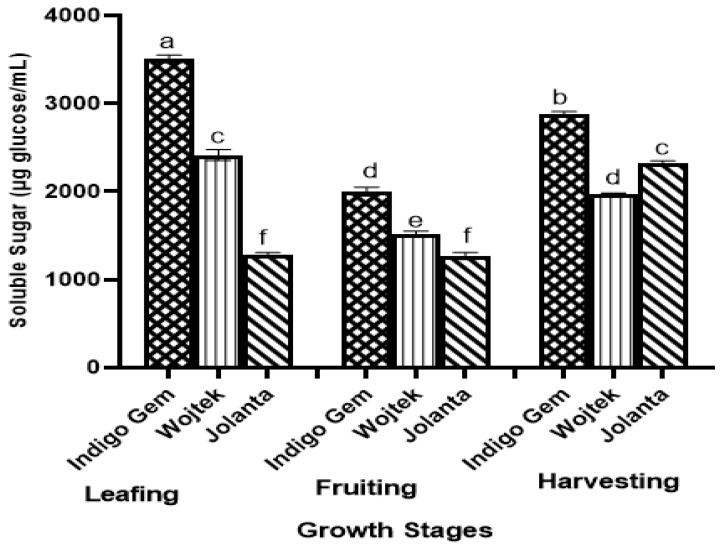
Soluble sugar (μg glucose/mL) of haskap leaf varieties at different growth stages. The different letters indicate a significant (*p* < 0.5) difference according to Tukey’s least significant difference (LSD) post hoc test.

Soluble solids (SSs), also known as the total soluble sugar of haskap leaves, were investigated. Our finding showed that SSs across the different varieties ranged from 1283.33 to 3507.03, 1269.64 to 1999.76, and 1977.11 to 2885.61 μg glucose/mL for the leafing, fruiting, and harvesting stages, respectively (Figure 1). Indigo gem had an average of 3507.03, 1999.76, and 2885.61 μg glucose/mL during the leafing, fruiting, and harvesting stages, respectively. Approximately a 43% decrease was recorded during fruiting and an 18% decrease during harvesting when compared with the SS concentration in Indigo Gem during the leafing stage. For Wojtek, a 37% decrease was observed during fruiting, and approximately 18% decrease during harvesting compared to the SS concentration at the leafing stage. The Jolanta variety, however, followed a different trend, with a 1% decrease during the fruiting stage and a 47% increase during the harvesting stage. No significant difference was observed between Jolanta at the leafing and fruiting stages.

### 3.2. Polyphenols in Haskap Leaves

**Figure 2:**
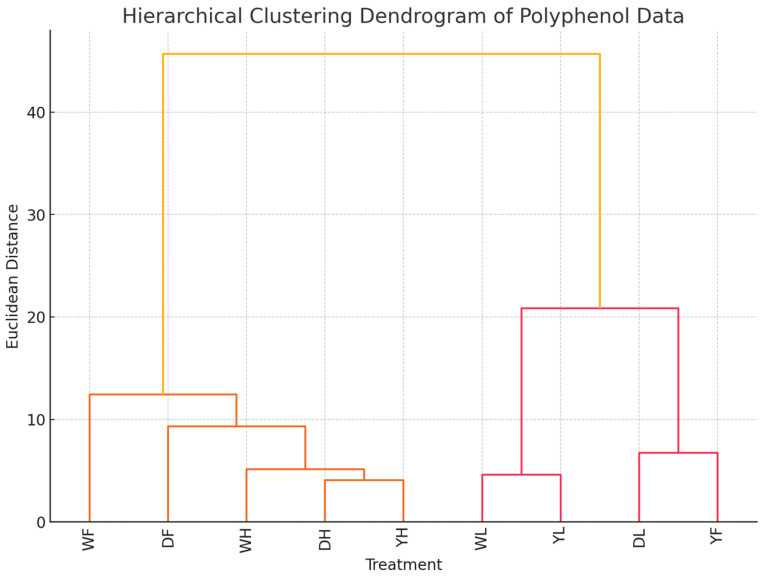
Hierarchical clustering dendrogram of polyphenols from different varieties of haskap leaf. DL, DF, and DH represent Indigo Gem at the leafing, fruiting, and harvesting stages of growth, respectively. YL, YF, and YH represent Wojtek at the leafing, fruiting, and harvesting stages of growth, respectively. WL, WF, and WH represent Jolanta at the leafing, fruiting, and harvesting stages of growth, respectively.

The hierarchical clustering dendrogram (Figure 2) was used to provide deeper insight into the relationships among the different combinations of haskap varieties and growth stages. A lower depth (closer clustering) of two varieties suggests that these varieties have some similar polyphenol profiles, irrespective of the stage of growth. By contrast, when varieties connect at a higher distance, this indicates more substantial differences in polyphenol composition between them. The harvesting stage of growth showed more influence across the three varieties of haskap leaf, as there was close clustering with lower depth.

**Figure 3:**
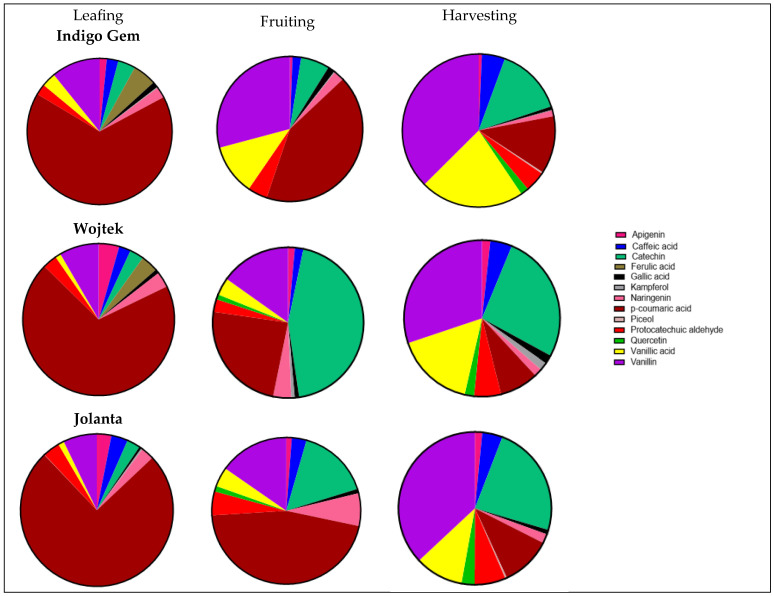
Polyphenol distribution (μM/mg) of Indigo Gem leaf, Wojtek leaf, and Jolanta leaf at the leafing, fruiting, and harvesting stages.

Thirteen polyphenols were identified in all the different varieties of haskap leaf (Figure 3), including three hydroxycinnamic acids, two flavonols, two hydroxybenzoic acids, two phenolic aldehydes, one flavone, one flavanone, one flavanol, and one di-hydroxybenzoic acid derivative. p-Coumaric acid had the highest percentage concentration across the different varieties of haskap leaf during the first growth stage (leafing), with Jolanta having the highest (75%), followed by Wojtek (70%), and Indigo Gem had the lowest concentration of about 66%. Catechin increased in percentage concentration across all growth stages for Indigo Gem (4% to 14%) and Jolanta (3% to 24%).

However, the reverse was the case in Wojtek, with an increase in catechin from 3% at the leafing stage to about 44% at the fruiting stage, then a decrease to 27% at the harvesting stage. Vanillin and vanillic acid were also observed to increase in percentage concentration as the plant developed from the leafing to the harvesting stage in the different varieties of haskap leaf. For Indigo Gem, the vanillin concentration increased from approximately 11% (leafing) to 37% (harvesting); the vanillin concentration increased from 8% (leafing) to 30% (harvesting) for Wojtek and from 7% (leafing) to 37% (harvesting) for Jolanta. For vanillic acid, an increase from 3% at the leafing stage to 22% at the harvesting stage was recorded for Indigo Gem, an increase from 1% at the leafing stage to 16% at the harvesting stage was recorded for Wojtek, and an increase from 1% at the leafing stage to 10% at the harvesting stage was recorded for Jolanta. Quercetin was first recorded at the fruiting and harvesting stages for Wojtek and Jolanta, while it was observed only at the harvesting stage for Indigo Gem.

### 3.3. Organic Acids in Haskap Leaves

**Figure 4:**
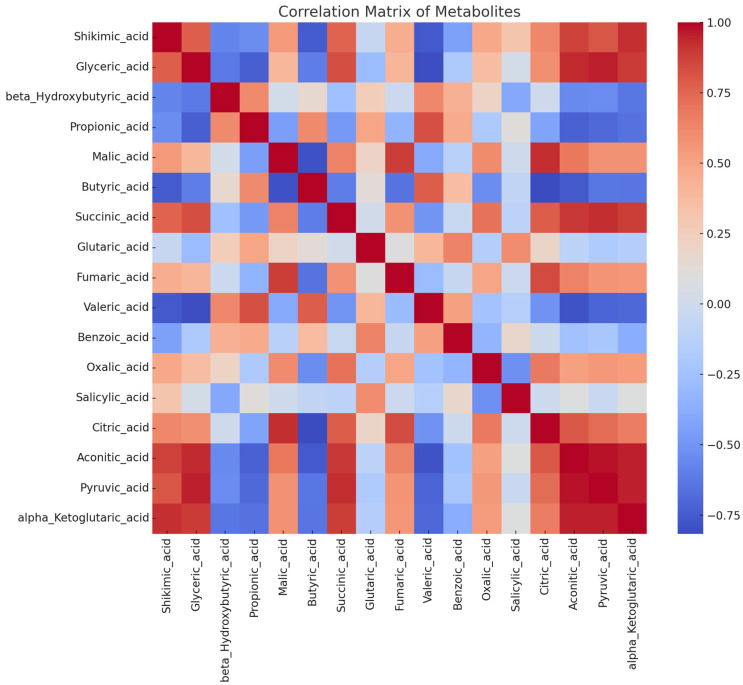
Heatmap of organic acids from haskap leaf varieties showing the relationship between different metabolites based on their correlation coefficients. The red color indicates a positive correlation, while the blue color indicates a negative correlation.

**Figure 5:**
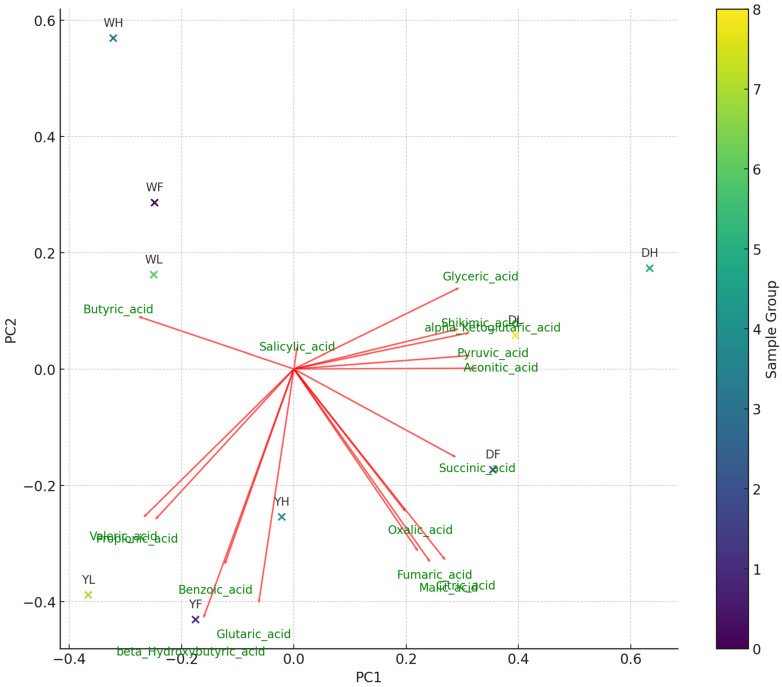
Principal component analysis (PCA) illustrating the relationship between variable organic acids (metabolites) and growth stages and varieties of haskap leaf. DL, DF, and DH represent Indigo Gem at the leafing, fruiting, and harvesting stages of growth, respectively. YL, YF, and YH represent Wojtek at the leafing, fruiting, and harvesting stages of growth, respectively. WL, WF, and WH represent Jolanta at the leafing, fruiting, and harvesting stages of growth, respectively.

A correlation heat map and PCA (Figure 4 and Figure 5) analyses were used to show the association among the variables (organic acids) across the different varieties of haskap leaf and the relationship across the growth stages. This interaction helps reveal important physiological insights in relation to specific plant behaviors and environmental responses. Correlation between metabolites might reflect underlying biochemical pathways that are active or suppressed during different growth stages or among varieties. The strong positive correlations between citric acid—malic acid (r = 0.95) and succinic acid—pyruvic acid (r = 0.88) might indicate that these organic acids were co-produced as part of the same metabolic pathway. Organic acids that are grouped together indicate a positive correlation (Figure 5), whereas those that are separated represent a negative correlation. In addition, organic acids clustered with a specific variety at a specific growth stage indicated that the growth stage and the variety influenced them, while those located further away from the variety and growth stage suggested a lack of influence from it. The PCA indicated that Indigo Gem at the leafing stage had a positive influence on organic acids like glyceric acid, shikimic acid, alpha-ketoglutaric acid, pyruvic acid, and aconitic acid.

A total of 17 organic acids were identified in different varieties of haskap leaf in this study, including 7 aliphatic acids (propionic, malic, butyric, succinic, fumaric, oxalic, and citric), 2 aromatic acids (shikimic and benzoic), 2 di-carboxylic acids (glutaric and alpha-ketoglutaric), 1 carboxylic acid (salicylic), 1 alkyl-carboxylic acid (valeric), 1 alpha-ketone acid (pyruvic), 1 tri-carboxylic acid (aconitic), and 1 hydroxy-carboxylic acid (beta-hydroxybutyric). During the leafing stage, the highest concentration of malic acid was recorded for Wojtek (472 μM/mg), while Jolanta had the lowest (362 μM/mg). A reduction of about 36%, 58%, and 48% was observed for Indigo Gem, Wojtek, and Jolanta, respectively, during the fruiting stage. However, at a later stage of growth (harvesting), there was a slight decrease in the concentration of malic acid by approximately 5%, 12%, and 15% for Indigo Gem, Wojtek, and Jolanta, respectively, when compared with the leafing stage. The following was the order from highest to lowest concentration across variety and developmental stage: malic > glyceric > oxalic > succinic > citric > aconitic > propionic > butryric > fumaric > pyruvic > alpha-ketoglutaric > valeric > benzoic > beta-hydroxybutyric > salicylic > glutaric acid.

### 3.4. Amino Acids in Haskap Leaves

**Figure 6:**
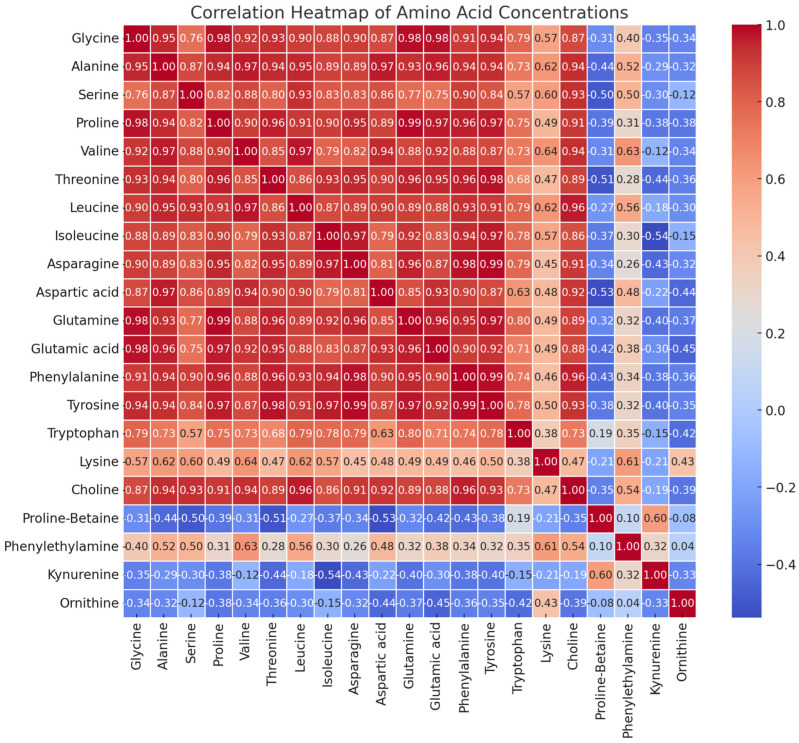
Pearson correlation matrix of amino acids from haskap leaf in response to varieties and growth stages combination. The red color indicates a positive correlation, while the blue color indicates a negative correlation.

**Figure 7:**
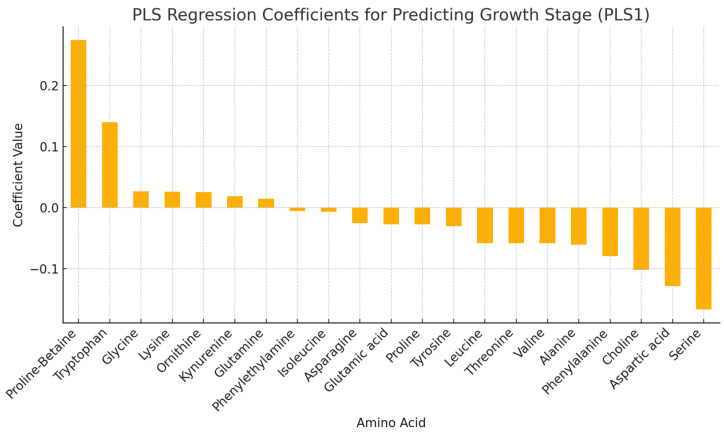
Partial least squares (PLS) regression coefficient for predicting amino acids profile at different growth stages.

The correlation heatmap and partial least squares (PLS) regression (Figure 6 and Figure 7) were used to understand the inter-relationships among the amino acids and identify the amino acid profile that best predicts or contributes to the growth stage of haskap leaves. Amino acids with larger absolute values of PLS 1 coefficients are most influential, whereas amino acids with smaller coefficients may vary less across stages of growth. This may imply that those amino acids are less distinctive in separating growth stages. Serine and alanine are significant potential biomarkers for identifying specific growth stages. This is an asset in agricultural or nutritional assessments. Essential amino acids showed strong and positive correlations (r = 0.8–0.99), except tryptophan (r = 0.5–0.79) and lysine (r = 0.38–0.64). The amino acids proline-betaine, phenylethylalanine, kynurenine, and ornithine showed very weak and negative correlations with other amino acids.

A total of seven essential amino acids (isoleucine, leucine, lysine, phenylalanine, threonine, tryptophan, and valine), nine non-essential amino acids (alanine, asparagine, aspartic acid, glutamic acid, glutamine, glycine, proline, serine, and tyrosine) and seven amino acid derivatives (phenylethylamine, orthenine, kynurenine, choline, proline-betaine, citrulline, and methionine-sulfoxide) were found in the different varieties of haskap leaf. Across all the different varieties of haskap leaf, non-essential amino acids—alanine, serine, glutamine, aspartic acid, and glutamic acid—showed the highest concentration at any given growth stage. By contrast, the essential amino acids valine, threonine, and phenylalanine showed the highest concentration for all three varieties of haskap leaf. Wojtek had the highest total essential amino acids (TEAAs) and total non-essential amino acids (TNEAAs), at 43.74 μM/mg and 455.01 μM/mg, respectively. The lowest concentration was recorded for Indigo Gem at 26.78 μM/mg (TEAAs) and 220.33 μM/mg (TNEAAs). However, across the growth stages, Jolanta showed the highest reduction in concentration of TNEAAs (90%) during harvesting, while Wojtek showed the highest reduction for TEAAs (89%) during fruiting.

## 4. Discussion

### 4.1. Soluble Sugar (SS) Content of Haskap Leaves at Different Growth Stages

The SS concentration of haskap leaves was significantly affected by growth stages. SS and titratable acidity contents are important fruit quality indices, and their ratio determines the flavor of fruits [18]. The higher the SS, the lower the titratable acidity in fruits, which increases toward the end of the harvesting stages. However, the reverse was the case with the leaves in the present study. The highest concentration of SS (3507.03 μg glucose/mL) was recorded at the beginning of the growth stage (leaf-out) and decreased during the fruiting stage, but it later increased slightly at the harvesting stage. The decrease in SS during the flowering/fruiting stage could be an indication that some content was being used up for metabolic processes, translating into the sweet taste of a ripe haskap fruit. Haskap berry fruits are high in soluble sugars. Senica et al. [9] recorded sugar in haskap fruit to be approximately 2.6 g/100 g, with glucose and fructose constituting most of the soluble sugar. A similar trend was observed in a recent study by Gamble et al. [19], who also concluded that haskap genotypic characteristics have a strong influence on fruit quality, which conforms to the results for the leaves of the different varieties in the present study. For example, the Indigo Gem variety, which has the highest recorded soluble sugar concentration, is categorized as the haskap hybrid with the sweetest berry amongst haskap varieties.

### 4.2. Polyphenol Concentrations of Haskap Leaves at Different Growth Stages

Polyphenols are a class of natural compounds with a wide range of chemical structures synthesized by plants. There are three major classes of polyphenols, which include phenolic acids, flavonoids, and non-flavonoids. Polyphenols are regarded as a group of biologically active compounds in plant-based foods involved in the protection of human health against several chronic diseases [20,21]. They are found in the human diet in foods such as fruits, nuts, seeds, vegetables, cereals, and beverages. Significant evidence has shown a positive correlation between polyphenols and a reduced risk of cardiovascular diseases, neurogenerative diseases, cancer, diabetes, atherosclerosis, low-density lipoprotein, and osteoporosis [22,23,24]. p-Coumaric acid dominated in all the varieties, as high as 35.06 μM at the early stage of haskap growth (leafing) and in two of the three varieties (Indigo Gem and Jolanta) at the fruiting stage. p-Coumaric acid is a hydroxycinnamic acid naturally occurring in whole-grain cereals, fruits, and vegetables as a hydroxy derivative of cinnamic acid [25]. It is classified as a nutraceutical and phytochemical due to its significant antimicrobial and anticancer activity [26,27,28]. Studies have also reported that p-coumaric has a protective effect against colon cancer in mammalian cells and exhibits anti-tumor activity in human malignant tumors [29,30]. However, in a study by Mattew et al. [31], p-coumaric exhibited a much lower scavenging capacity for radicals of 1,1-diphenyl-2-picrylhydrazyl and superoxide and a lower reducing power than caffeic and ferulic acids. Ferulic acids with similar physiological activities as p-coumaric were, however, scanty in the haskap leaves investigated in this study. They were identified only in Indigo Gem and Jolanta at the leafing stage of haskap. Ferulic acid is an important structural component of plant cell walls that gives rigidity and vigor to them. They are naturally found in the cell walls of cereals such as rice endosperm, maize bran, and wheat straws, which are considered agricultural by-products [32]. Caffeic acid was observed to be present in all varieties of haskap leaves at all stages of growth; it decreased slightly during the fruiting stage and later increased during the harvesting stage. Caffeic acid is a cinnamic acid that is considered an important phenylpropanoid found in plants. Phenylpropanoids are known for their role in plant stress tolerance by the preservation of the plant’s metabolic machinery and ultrastructure from stress-induced oxidative alterations [33,34]. Caffeic acid has a strong antioxidant ability by donating hydrogen to stabilize and neutralize the free radicals released during plant stress [35]. This could be why few pests and diseases are known to afflict haskap plants cultivated under field conditions [36]. Gallic acid and protocatechuic aldehyde are the hydroxybenzoic acids identified in haskap leaf varieties in the current study. Protocatechuic acids showed an increase in concentration as growth progressed. The opposite trend was observed for gallic acid in Indigo Gem, which showed a decrease in concentration at the harvesting stage. Protocatechuic aldehyde, a precursor of protocatechuic acids, is widely distributed in plants, fruits, and vegetables. In a recent review study by Widy-Tysz-Kiewicz [37], both protocatechuic acid and protocatechuic aldehyde influenced the gut microbiota profile, which contributes to the improvement of human health. Their consumption has been linked to lowered risk of some chronic diseases, such as cardiovascular and Alzheimer’s disease and cancer.

Kaempferol and quercetin are the flavonols identified in haskap leaves. They are the most common flavonols found in food [38]. Kaempferol is often associated with quality grapes and wines. It has great potential for the prevention and treatment of human diseases such as cancer, diabetes, neurogenerative diseases, and obesity. Quercetin is likely the most researched polyphenol due to its relatively high bioavailability [39]. It is widely present in fruits, vegetables, and many dietary supplements and herbal remedies in the form of quercetin glycosides [40]. Quercetin has been reported to possess excellent antioxidants in many studies and has anti-cancer, anti-inflammatory, ant-ulcer, anti-proliferative, and anti-allergic effects [41,42,43]. Dawson [36] earlier reported an appreciable concentration of quercetin-3-glycoside in the leaves of two haskap varieties. However, the focus was on the effect of fruit development on the secondary metabolites in haskap fruits and leaves. Quercetin’s earliest sign was at the fruiting stage, which increased in concentration as haskap plant growth advanced.

Catechin belongs to the flavanol class of polyphenols commonly found in the leaves of *Camellia sinensis* [44,45,46] and other sources, such as grapes, apples, pears, and cherries. Catechin is one of the top-researched polyphenols in epidemiological and experimental studies in connection with the treatment of the top three ailments: cancer, cardiovascular diseases, and aging. Both in-vitro and animal studies have shown a strong inverse correlation between catechin intake and the risk of mortality due to coronary heart diseases and degenerative diseases [47,48]. Although they are not considered essential nutrients to humans, a daily intake of 18–50 mg/day can help improve human health by preventing various diseases. The catechin concentration increased from 1.19 to 12.41 μM/mg as haskap growth advanced. However, the highest concentration was recorded at the fruiting stage, with Wojteck having the highest (12.41 μM/mg). Kwits et al. [49] reported that catechins in green and black tea are rapidly absorbed in water. Haskap leaf could be a cheap alternative source of raw material for herbal tea production.

Other polyphenols of significant concentrations identified in haskap leave are vanillic acid and vanillin. It was observed that vanillin and vanillic acid concentrations increased consistently as the growth stage advanced. Vanillin is an important flavor and aroma compound from the bean of the vanilla orchid (*Vanilla planifolia*). It is one of the most widely used natural flavoring materials worldwide. Vanillin has been reported to show strong antioxidant and antimicrobial properties [50,51]. The oxidation of vanillin produces vanillic acid, which is also regarded as a derivative of benzoic acid. Vanillic acid is used as a flavoring and preservative agent. It is naturally found in food such as whole grains, fruits, green tea, herbs, juices, and wines. Vanillic acids have been isolated abundantly in black sesame pigment, which is used as a food supplement in the prevention of Alzheimer’s disease. This is attributed to its numerous pharmacological properties, such as antioxidant, anti-apoptotic, neuroprotective, hepatoprotective, cardioprotective, anti-inflammatory, and immuno-stimulating properties.

### 4.3. Organic Acid Concentrations of Haskap Leaves at Different Growth Stages

Organic acids are compounds classified by the number of carboxylic functions with acidic properties. They constitute the second most abundant soluble solid components in fruits and are responsible for the tart flavor of fruit juice. Organic acids play an important role in various metabolic and catabolic pathways in both plants and animals. They have antioxidant, antibiotic, and antimicrobial properties [52]. Organic acids are generally recognized as safe (GRAS) and used as food preservatives.

Research has often shown that different varieties of the same species can have significantly varied metabolic profiles, which might be a response to genetic differences and adaptation strategies. In addition, growth stages can also influence metabolic profiling. The different growth stages represent critical transitions in plant metabolism, where metabolic shifts occur to support each stage’s physiological demands. For example, citric acid and malic acid are often more abundant in leafing stages to support initial energy demands. This is evident in the smaller concentration recorded during fruiting compared to the leafing stage. As the haskap fruit developed, the organic acid concentration of the haskap leaves decreased. The most common organic acids in fruits and vegetables are malic, citric, isocitric, galacturonic, quinic, oxalic, and tartaric acids. In this study, malic acid (188–473 μM/mg) dominated the organic acids in the haskap leaves at all stages of growth. Malic acid has been reported to be the second predominant organic acid in different varieties of haskap berries [53,54]. The concentrations of citric acids obtained in this work are lower than those reported in the berries by the same authors. Citric acid dominated the organic acids in the berries. This could be responsible for the tart taste experienced in haskap berries. It was observed that most of the organic acids decreased significantly during the fruiting stage. This could be an indication that organic acids play an important role in some metabolic activities during fruit development, hence the reduction in concentration.

### 4.4. Amino Acid Concentrations of Haskap Leaves at Different Growth Stages

This is the first time amino acid concentration in haskap leaves has been analyzed. Sonchor et al. [55] investigated the distribution of amino acids in 19 cultivars of haskap berries. The authors reported that aspartic acid predominated, while alanine was less abundant in all the 19 cultivars studied. In this study, nonessential amino acids predominated in haskap leaves, while some essential amino acids were observed below the detection limit. Glutamine, glutamic acid, alanine, aspartic, and serine were the amino acids with the highest concentrations. Plant-based foods are not the best source of amino acids; however, haskap leaves have the potential to provide some essential nutrients to consumers. Interestingly, it was observed that haskap leaves contain a high concentration of choline (26.30–173.00 μM/mg) and an appreciable quantity of amino-acid metabolites at all stages of growth, with the leafing stage having the highest concentration. Choline is recognized as a vital amine for maintaining adult health [56,57,58]. Recent work has shown its role in brain development during fetal development. Studies have shown the development of liver damage in healthy male humans on choline-deficient diets. This resulted from elevated plasma alanine aminotransferase due to diminished plasma choline and phosphatidylcholine concentrations [56]. Choline also helps with depression and muscle control and regulates memory. Kynurenine is a metabolite of the amino acid L-tryptophan, which plays a critical role in the kynurenine pathway [59]. The kynurenine pathway is key in generating cellular energy in the form of nicotinamide adenine dinucleotide (NAD+).

## 5. Conclusions

The results obtained indicate that the growth stages of the haskap plant have a strong influence on its nutrients and polyphenol concentrations. The leafing stage just before the onset of fruiting could be the best time to harvest leaves with the highest concentration of nutrients and polyphenols. The study shows that haskap leaves at the leafing growth stage have the potential to be explored as a raw material in simple infusions for medicinal use or for processing brewed products. However, future toxicology studies are needed to ascertain their safety for human consumption.

## Data Availability

The original contributions presented in the study are included in the article, further inquiries can be directed to the corresponding authors.
